# Prophylactic and therapeutic effects of *Limosilactobacillus reuteri *E on 5-fluorouracil-induced intestinal mucositis in Caco-2 cells

**DOI:** 10.1007/s12223-025-01310-3

**Published:** 2025-08-28

**Authors:** Barbora Hlubinová, Hana Kiňová Sepová, Ľudmila Pašková, Peter Novák, František Bilka, Hana Drahovská, Tomáš Szemes, Andrea Bilková

**Affiliations:** 1 Department of Cell and Molecular Biology of Drugs, Faculty of Pharmacy, Comenius University Bratislava, Bratislava, Slovakia; 2 Department of Molecular Biology, Faculty of Natural Sciences, Comenius University Bratislava, Bratislava, Slovakia; 3 Science Park, Comenius University Bratislava, Bratislava, Slovakia

**Keywords:** *Limosilactobacillus reuteri* E, Intestinal mucositis, 5-Fluorouracil, Caco-2, Probiotics

## Abstract

This work studied the potential of probiotic candidate *Limosilactobacillus reuteri* E to ameliorate intestinal mucositis in vitro. By PathogenFinder analysis of genome sequence, the strain was not predicted to be pathogenic. Genes responsible for adhesion (MapA, EF-Tu) and immunomodulation (e.g., EpsD, EpsF, EpsC, enzymes responsible for the synthesis of exopolysaccharides) abilities were localized in the strain genome. In the co-cultivation model of Caco-2 cells and *L. reuteri* E, lactobacilli up-regulated gene expression of occludin and claudin 1 (tight junction proteins) and paralemmin (receptor-like molecule) in Caco-2. The prophylactic and therapeutic potential of *L. reuteri* E to modulate intestinal mucositis was investigated by its administration to Caco-2 cells before and after 5-FU treatment. In bacteria, both prophylactic and therapeutic approaches resulted in significantly higher levels of EpsF glycosyltransferase gene expression (1.5-fold and 3.4-fold, respectively) compared to lactobacilli co-cultivated with untreated Caco-2 cells. In Caco-2 cells, significantly increased gene expression of occludin and claudin 1 in prophylaxis was observed. Moreover, lactobacilli used in prophylaxis ameliorated 5-FU-induced toxicity in Caco-2 cells, as shown by increased cell viability. This may be related to improved anti-inflammatory-to-pro-inflammatory ratio (higher A20 and IL-10 vs. IL-1β and IL-8 gene expression) observed in prophylactic conditions compared to the therapeutic lactobacilli application to Caco-2 cells. The results obtained suggest that *L. reuteri* E may be more effective in the prophylaxis of 5-FU-induced intestinal mucositis than in therapeutic use.

**Supplementary Information:**

The online version contains supplementary material available at https://doi.org/10.1007/s12223-025-01310-3.

## Introduction

Administration of 5-fluorouracil (5-FU), an analogue of uracil, as a chemotherapeutic agent causes intestinal mucositis (IM) as a serious side effect in 50–80% of patients suffering from various cancer types. This significantly impacts their quality of life and causes a delay in the treatment cycle, therefore reducing the overall efficacy of the treatment (Bowen et al. 2006; Kim et al. 2015).

Several studies have shown that chemotherapy alters the composition of the gut microbiota and can lead to dysbiosis (van Vliet et al. 2010; Montasier et al. 2015). Montoori et al. (2017) described decreased levels of lactobacilli after 5-FU treatment. Therefore, one of the perspective approaches to prevent or treat IM seems to be the administration of probiotics (Batista et al. 2020). Probiotics are defined as “live microorganisms which when administered in adequate amounts, confer a health benefit on the host” (Hill et al. 2014). Lactobacilli represent one of the most significant groups of them.

Among the probiotic bacteria, strains of *L. reuteri* are widely studied for their ability to prevent and treat gastrointestinal disorders (Luo et al. 2023; Lee et al. 2025), but their role in inflammatory conditions such as chemotherapy-induced mucositis has not yet been clearly defined. Recently, it has been shown that strains of *L. reuteri* can protect and strengthen the intestinal barrier by maintaining the integrity of tight junction (TJ) proteins and by induction of mucin production (Dudík et al. 2020; Jiang et al. 2023). However, the potential beneficial effects of probiotic microorganisms are diverse, heterogeneous, and dose-dependent. Therefore, it is necessary to characterize each potential probiotic strain and research its effects and mechanisms of action in different pathological conditions for both prophylaxis and therapy (Plaza-Díaz et al. 2017). One of the promising bacterial products with anti-inflammatory and antitumour activity seems to be exopolysaccharides (EPS) (Kang and La Pointe 2018). In addition, it was demonstrated that adhesion molecules, such as mucus binding protein, may also serve as immunomodulatory factors (Li et al. 2014).

To predict the properties and health-promoting effects of probiotic strains, there has been an increasing focus on bioinformatic in silico analysis of their whole genome sequences (WGS)—probiogenomics (da Silva et al. 2024). Such studies may serve as the base for further in vitro and in vivo experiments.

There are many studies that focus on the effects of probiotic microorganisms on human cells both in vitro and in vivo, but few studies look at this problem from the opposite perspective, i.e., what happens inside the microbial cell. Therefore, we investigated the responses of both prokaryotic and eukaryotic cells in order to understand how these different cells respond to mutual stimuli.

The aim of the present study was to investigate the whole genome sequence of *L. reuteri* E and identify and describe genes possibly responsible for interaction with Caco-2 human intestinal cell line. Subsequently, mRNA expression of selected lactobacilli adhesion and immunomodulatory molecules was surveyed in in vitro co-cultivation model before and after 5-FU administration to Caco-2 cells. In the same experimental setup, we studied gene expression of TJ proteins and pro- and anti-inflammatory cytokines in Caco-2 cells untreated and treated by 5-FU, and the effect of *L. reuteri* E, administered in a prophylactic and therapeutic way on this process.

## Materials and methods

### Origin and cultivation conditions of *Limosilactobacillus reuteri* E.

*Limosilactobacillus reuteri* E, a potential probiotic strain isolated from the stomach mucosa of breast-fed lamb (breeding station Očová, Slovakia) identified (Bilková et al. 2008) and characterized before (Bilková et al. 2011; Kiňová Sepová et al. 2011; Kiňová Sepová and Bilková 2013; Májeková et al. 2015; Greifová et al. 2017; Dudík et al. 2020), was cultivated 18 h in de Man, Sharpe and Rogosa (MRS; VWR, USA) broth anaerobically at 37 °C.

Bacteria were washed twice with saline and adjusted to A_600_ ~ 0.1 in the pure Dulbecco’s Modified Eagle’s Medium (DMEM; Sigma-Aldrich, Germany) before co-culture tests.

### Bacterial whole genome sequencing, assembling, and annotation

*L. reuteri* E genomic DNA was purified with a Higher Purity™ Bacterial Genomic DNA Isolation Kit (CanvaxBiotech, Cordoba, Spain), and DNA was quantified using a Qubit™ dsDNA HS Assay Kit (Thermofisher Scientific, Eugene, USA). Sequencing libraries were prepared using the Nextera XT DNA Library Prep Kit protocol (Illumina, San Diego, USA) and purified on AMPure XP magnetic beads (Beckman Coulter Life Sciences, Indianapolis, USA). The quality of the library was checked using a high-sensitivity DNA electrophoresis chip and a 2100 Bioanalyzer Instrument (Agilent Technologies, Santa Clara, USA). Sequencing was carried out with 2 × 150 bp reads using the Illumina NextSeq 500™ platform (Illumina). Finally, 3,740,146 raw reads and 3,055,016 deduplicated reads were obtained.

Adapters and low-quality ends of sequenced reads were removed using Trimmomatic (v0.36; Bolger et al. 2014); fragments without sufficient length of both reads (> 45 bp) were removed; the reads were deduplicated using FastUniq (v1.1; Xu et al. 2012) and finally assembled de novo using SPAdes (v3.10.1; Bankevich et al. 2012).

The sequence was annotated by using Bacterial and Viral Bioinformatics Resource Center (BV-BRC; Wattan et al. 2017) and Rapid Annotation using Subsystem Technology (RAST, v2.0; Aziz et al. 2008). Genomic DNA was screened for specific genes associated with probiotic properties. For searching of potential pathogenicity and virulence factors, PathogenFinder 1.1 (default parameters were used; Cosentino et al. 2013) and BV-BRC_VF and Victors (both at BV-BRC portal) were used, respectively.

Bioinformatic tools used in this analysis are well-designed and user-friendly, offering a wealth of valuable information applicable to the interpretation of genomic data.

### Cultivation of Caco-2 cells

The human colon adenocarcinoma Caco-2 cell line (cat. number 300137; Cell Line Service, Germany) was cultivated in enriched DMEM medium containing 4.5 g/L glucose and supplemented with 1% streptomycin, 1% penicillin, and 10% fetal bovine serum (all Sigma-Aldrich, Germany) under standard conditions, 37 °C, 5% CO_2_ (humidified incubator Memmert, Germany). Cells were grown in plastic culture plates Ø 100 mm (Sarstedt, Germany) and were passaged using trypsin (1 × trypsin–EDTA solution; 0.05% (w/v); MPbio, USA) when confluent. Before experiments, Caco-2 cells were seeded in 12 (for gene expressions) or 24-well plates (for MTT tests) (Sarstedt, Germany).

### MTT test

Caco-2 cells at density 0.5 × 10^6^ cells per well were seeded in a 24-well plate 48 h before the MTT (3-[4,5-dimethylthiazol-2-yl]−2,5-diphenyltetrazolium bromide) experiment.

#### Lactobacilli toxicity

Both time (2, 4 h) and dose effect (bacterial saline suspensions 1 × 10^6^ CFU per mL, 5 × 10^6^ CFU per mL, 1 × 10^7^ CFU per mL, 5 × 10^7^ CFU per mL, 1 × 10^8^ CFU per mL) of *L. reuteri* E on Caco-2 cells viability were studied.

#### 5-FU toxicity

Time (2, 4, 24 h) and dose (25 µmol/L, 50 µmol/L, 100 µmol/L, 500 µmol/L, 1 mmol/L, 2 mmol/L) effects of 5-FU (Accord, GB) cytotoxicity on Caco-2 cells were studied. The 5-FU concentrations and exposure times tested were selected according to Fang et al. (2014) and Cheah et al. (2014).

After optimization, lactobacilli (1 × 10^8^ CFU per mL) were co-cultivated for 4 h with Caco-2 cells before (prophylaxis) or after (therapy) exposure to 50, 100, and 500 µmol/L concentrations of 5-FU.

MTT (modified according to Bahuguna et al. 2017) was performed in pure DMEM medium with the corresponding tested agent (lactobacilli or 5-FU). Afterwards, Caco-2 cells were washed twice with PBS (Sigma-Aldrich, Germany). Subsequently, 0.5 mL of pure DMEM containing MTT (Sigma-Aldrich, Germany) was added to the cells in a concentration of 0.5 mg/mL for 4 h at 37 °C in the dark. After incubation, the medium was removed, and MTT crystals were dissolved in 1 mL of DMSO (Sigma-Aldrich, Germany). After 20 min incubation at laboratory temperature, the A_595_ was measured (Epoch microplate spectrophotometer, Biotek, USA). Viability was expressed as a percentage of viable cells in comparison to control (100%).

Co-cultivation of *L. reuteri* E with Caco-2 cells for gene expression studies.

Lactobacilli (1 × 10^8^ CFU per mL) were co-cultivated for 4 h with Caco-2 cells in pure DMEM medium in 12-well plates before (prophylaxis) or after (therapy) 5-FU exposure at a dose of 100 µmol/L (37 °C, 5% CO_2_, humidified atmosphere; Memmert, Germany). In experiments investigating the effect of lactobacilli on Caco-2 cells, untreated Caco-2 cells were used as a control. In experiments investigating the prophylactic or therapeutic effect of lactobacilli on 5-FU-treated Caco-2 cells, Caco-2 cells treated with 100 µmol/L 5-FU were used as a control.

### Bacterial RNA isolation

*L. reuteri* E total RNA was isolated using a commercial kit PureLink™ RNA Mini Kit (Invitrogen, USA). Isolated RNA in an amount of 500 ng was reversely transcribed into cDNA using PrimeScript RT reagent kit (Takara, Japan) according to the manufacturer’s instructions. Reverse transcription was run in the thermocycler MasterCycler personal (Eppendorf, Germany) under reaction conditions: reverse transcription 37 °C for 15 min, reverse transcriptase inactivation 85 °C for 5 s, cooling 4 °C.

### Caco-2 cells RNA isolation

Caco-2 total RNA was isolated using the RNazol (Molecular Research Center, USA) followed by RNA reverse transcription into cDNA using the same procedure and kit as stated in the chapter “Bacterial RNA isolation”.

### Quantitative PCR

qPCR was performed according to Dudík et al. (2020) with qPCR mix (5 × HOT FIREPol® EvaGreen®qPCR Mix Plus, Solis Biodyne, Estonia). Reaction conditions were as follows: 95 °C for 15 min, followed by 40 cycles of 95 °C for 15 s, 60 °C for 30 s, and 72 °C for 30 s. All reactions were performed in duplicates and with a no-template control. The relative mRNA expression was analyzed using the ΔΔCt method (Winer et al. 1999). Primer sequences are listed in Table 1.

**Table 1:** Sequences of PCR primers

Target gene	Sequence Forward (5′ – 3′) Reverse (5′ – 3′)	Target purpose	Source
16S rRNA	TTTGGCTATCACTCTGGGA CCGAAACCCTTCTTCACTC	*L. reuteri* E	Bolado-Martínez et al. (2012)
EpsD	AGCCCAATTTCCGAACAGTTC GGTGACGGTTGACTTTCCTTC	*L. reuteri* E	This study
EpsF	ATGGGTCTACCACGCCATCA AGGCAACTTAGTCACCGCAT	*L. reuteri* E	This study
MapA	GCAGTTGCTAAAAAGATGGGCT CCCGTTCAGGTGTCTGTGTA	*L. reuteri* E	Dudík et al. (2020)
EF-Tu	GACGCTTTGGAGTTGGGATG ACGATTTCCCTGGTGACGAT	*L. reuteri* E	Dudík et al. (2020)
18S rRNA	TGTGATGCCCTTAGATGTCC GATAGTCAAGTTCGACCGTC	Caco-2	Dudík et al. (2020)
PRLM	GTCCATGCTGTGGACGG GTCCGCTTTGTGGATGAGTT	Caco-2	Matsuo et al. (2012)
OCLN	TTGGATAAAGAATTGGATGACT ACTGCTTGCAATGATTCTTCT	Caco-2	Akbari et al. (2015)
CLDN1	AGCTGGCTGAGACACTGAAGA GAGAGGAAGGCACTGAACCA	Caco-2	Akbari et al. (2015)
NF-κB	TCCCATCTTTGACAATCGTGC TGGTCCCGTGAAATACACCTC	Caco-2	This study
IL-1β	AAGCCATAAAAACAGCGAGGGA AGCAGCCTGTTGTGCCTTGT	Caco-2	This study
IL-8	CCTGATTTCTGCAGCTCTGTG TCCAGACAGAGCTCTCTTCCAT	Caco-2	This study
A20	GAAAGTCAGTCCCACAGCGT GGCTCGATCTCAGTTGCTCT	Caco-2	This study
IL-10	AAGCCTGACCACGCTTTCTA GCTCCCTGGTTTCTCTTCCT	Caco-2	Dudík et al. (2020)

*16S rRNA*, 16S ribosomal RNA; *EpsF*, exopolysaccharide biosynthesis glycosyltransferase *EpsD*; *EpsD*, tyrosine-protein kinase; *MapA*, mucus adhesion-promoting protein A; *EF-Tu*, elongation factor Tu; *18S rRNA*, 18S ribosomal RNA; *PRLM*, paralemmin; *OCLN*, occludin; *CLDN1*, claudin 1; *NF-κB*, nuclear factor κB; *IL-1β*, interleukin 1β; *IL-8*, interleukin 8; *A20*, tumor necrosis factor α-induced protein 3; *IL-10*, interleukin 10

### Statistical analysis

Results are expressed as arithmetic mean ± standard error of the mean. The data were analyzed using GraphPad Prism 9 (GraphPad, La Jolla, USA) statistical analysis. The unpaired Student’s t-test with Welch’s correction was used for statistical processing of the two groups’ results. The one-way ANOVA statistical method with Tukey’s multiple comparisons test was used when comparing three or more groups. The differences to be statistically significant are considered at *p* < 0.05, *p* < 0.01, *p* < 0.001, and *p* < 0.0001. All samples were analyzed in three parallels (n = 3). All graphs were prepared in GraphPad Prism 9.

## Results and discussion

Selected beneficial properties of the potentially probiotic strain *L. reuteri* E have been studied in the past. Among the three strains of lactobacilli isolated from lamb, *L. reuteri* E showed the strongest antimicrobial activity against ten Gram-positive and Gram-negative pathogenic bacteria tested by the streak line method (Bilková et al. 2011). This strain has been shown to be capable of producing antimicrobial substances such as organic acids (lactic, acetic, phenyllactic), ethanol, and reuterin in vitro. Of the biogenic amines that have immunomodulatory potential, *L. reuteri* E has demonstrated the ability to produce tyramine (Kiňová Sepová and Bilková, 2013; Greifová et al. 2017). The strain has proved good survival in the presence of 0.5% and 1% bile salts. Based on the antibiogram, *L. reuteri* E was found to be resistant to ceftazidime, vancomycin, ofloxacin, and ciprofloxacin. However, sensitivity to ceftazidime was established after incubation in both bile concentrations (Májeková et al. 2015). Previous experimental results also showed that pre-cultivation of *L. reuteri* E in mucin-enriched medium increased its adhesion to HT-29 cells in vitro and increased mucin expression in these cells (Dudík et al. 2020). The safety of *L. reuteri* E was confirmed in a rat model in vivo (Marušáková et al. 2022). In this study, based on characterization and description of WGS and in silico analysis, we selected genes involved in the production of exopolysaccharides and *L. reuteri* E adhesion and monitored their expression before (prophylaxis) and after (therapy) exposure to 5-FU-treated Caco-2 cells.

According to Merenstein et al. (2023), genome analysis is one way to characterize a probiotic strain and confirm its safety. *L. reuteri* E genome was assembled into 85 contigs with 262 × average coverage depth. The WGS comprises 1,902,828 bp chromosome with N_50_ and L_50_ values of 44,693 and 12, respectively, and a content of G + C 38.7%. It contains 1946 coding genes, of which 1474 have been functionally evaluated in 215 subsystems, representing 31% of all annotated genes, and 472 hypothetical genes. There are 4 rRNA and 62 tRNA genes. WGS is deposited at GenBank under accession number JAHQZV000000000.1.

PathogenFinder 1.1 (Cosentino et al. 2013) was used for searching pathogenic families, and *L. reuteri* E was not predicted as a human pathogen (probability 0.09). Virulence factors were also predicted by the BV-BRC server. The two copies of the *rfb*A gene were the only virulence genes predicted by BV-BRC_VF and Victors in the *L. reuteri* E genome. The *rfb*A encodes glucose-1-phosphate thymidylyltransferase (EC 2.7.7.24; Supplemental Table S1) and is a part of the *rfb*ABCD cluster that is probably involved in dTDP-L-rhamnose biosynthesis (Zivkovic et al. 2015). From further in silico analysis, we assume that these gene products may be involved in EPSs production as well as in lactobacilli-host cell interactions, as discussed below.

Pathogenesis of IM is complex and involves different mechanisms, including intestinal barrier dysfunction. Inflammation and gut microbiota disorder contribute to the mucosal injury (Wu et al. 2020). It seems that some probiotic strains are able to prevent and/or treat 5-FU-induced damage both in vitro and in vivo (Fang et al. 2014; Justino et al. 2020; Marsova et al. 2020; Salek et al. 2024).

Prior to the experiments, we set up the conditions for the IM model by optimizing the concentration of 5-FU and the exposure time as well as the safe dose of lactobacilli. Based on the results of MTT tests, viability of 80% of Caco-2 was achieved at 100 µmol/L 5-FU acting for 24 h (data not shown). According to literature, reduction of Caco-2 cells viability to 70–85% after administration of 5-FU mirrors its gastrointestinal toxicity observed during chemotherapy in cancer patients (Cheah et al. 2014). The effect of lactobacilli on Caco-2 cells was investigated also by MTT test. The proper dose and exposure time of lactobacilli were analyzed. Although viability did not significantly decrease at any of the tested numbers of bacteria (1 × 10^6^ CFU per mL, 5 × 10^6^ CFU per mL, 1 × 10^7^ CFU per mL, 5 × 10^7^ CFU per mL, 1 × 10^8^ CFU per mL) and times of co-cultivation (2 h, 4 h), Caco-2 viability decreased slightly (not significantly) after 2 h of co-cultivation. No change in the viability of Caco-2 cells was observed after 4 h co-cultivation compared to the untreated control (data not shown). Therefore, the highest tested dose of bacteria (1 × 10^8^ CFU per mL) and exposure time of 4 h were used for the co-cultivation of *L. reuteri* E with Caco-2 cells in all further experiments.

**Fig. 1:**
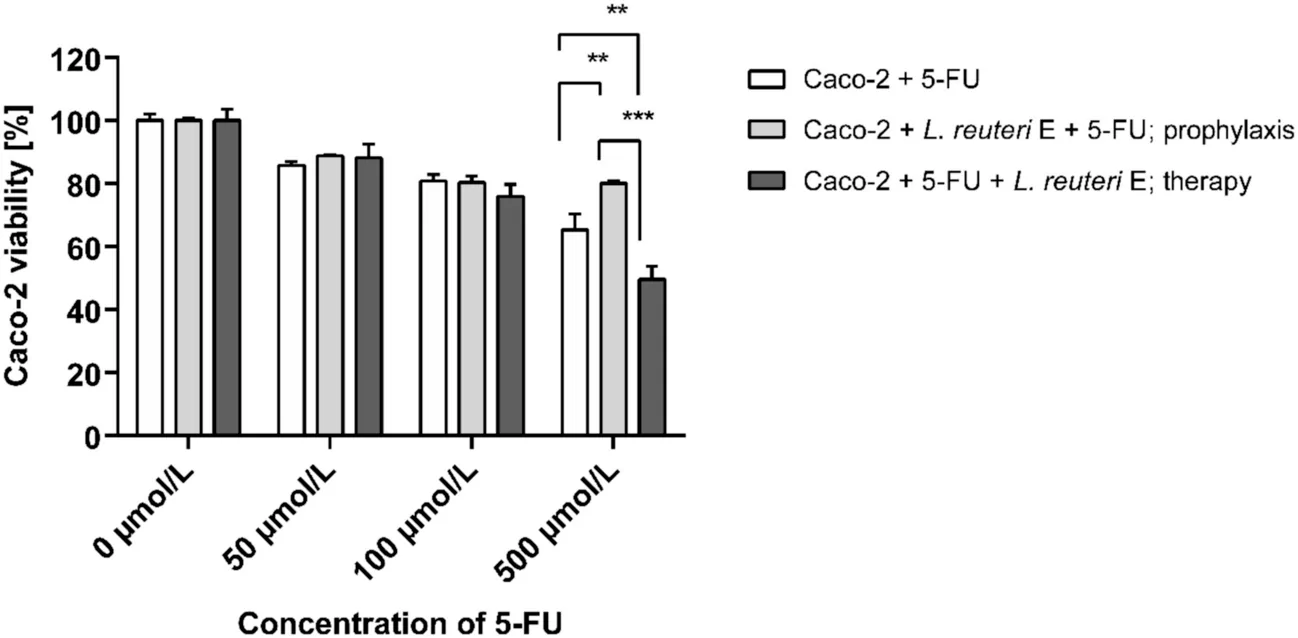
Prophylactic and therapeutic effect of *L. reuteri* E on Caco-2 cells treated by 5-FU. Data represent mean ± SD (*n* = 3). ** *p* < 0.01, *** *p* < 0.001

To test the potential of prophylactic and therapeutic administration of *L. reuteri* E to modulate 5-FU damage, changes in Caco-2 cell viability were estimated by the MTT assay. In addition to 100 µmol/L 5-FU, a lower and a higher concentration (50 and 500 µmol/L, respectively) were used (Fig. 1). *L. reuteri* E was effective in increasing the viability of Caco-2 only in prophylaxis manner of application and the highest 5-FU dose used. Surprisingly, when lactobacilli were used as therapy, Caco-2 cell viability decreased to 49.64% compared to the effect of 5-FU alone (65.29%). It seems that bacteria can act as an additional stress factor for 5-FU-treated Caco-2 cells. Intestinal cells were unable to cope with this additional stress, and it appears to potentiate the effects of 5-FU on cell viability.

To study the genes involved in prokaryotic-eukaryotic interaction, we focused on several targets. Within WGS of *L. reuteri* E, we identified several genes predisposing the strain to produce EPS. Many lactic acid bacteria can synthesize these extracellular polymers, which are attached to bacteria or secreted into the surrounding environment (Wang et al. 2010). They are known to have immunomodulatory, antitumor, and antioxidative activities (Salimi and Farrohk 2023). EPS can also affect colonization by lactic acid bacteria through interactions with the mucosa and prevent pathogenic biofilm formation (Mays et al. 2020). The antimicrobial activity of EPSs acts directly by stimulating growth and biofilm formation of commensal and probiotic bacteria, and indirectly by stimulation of the host immune response (Werning et al. 2022). EPSs produced by lactic acid bacteria are able to modulate both humoral, including tumor necrosis factor α, interleukins 1β, 2, 6, 10, and 12 (Wang et al. 2018; Saadat et al. 2019; You et al. 2020), and cellular, e.g., natural killer cells, T and B lymphocytes, phagocytes (Saadat et al. 2019; Angelin and Kavitha 2020) components of the immune system. EPS gene clusters in lactobacilli showed high variability depending on the natural habitat of the strain. According to genome analysis of lactobacilli, it was proven that for production of EPSs, genes encoding regulation enzymes, glycosyltransferases, repeating units’ biosynthesis, determination of chain length, and enzymes responsible for polymerization and export of repeating units are necessary (Deo et al. 2019). Several of these genes were identified in the *L. reuteri* E genome (Supplemental Table S1). Some of them were chosen for further studies.

To investigate the effect of 5-FU damaged Caco-2 cells on *L. reuteri* E immunomodulation and adhesion potential, we determined the expression of several genes participating in these processes (immunomodulation: EpsD, EpsF; adhesion: mucus adhesion-promoting protein A (MapA) and elongation factor Tu (EF-Tu)) after their co-cultivation with Caco-2 cells before (prophylaxis) and after (therapy) 5-FU administration.

**Fig. 2:**
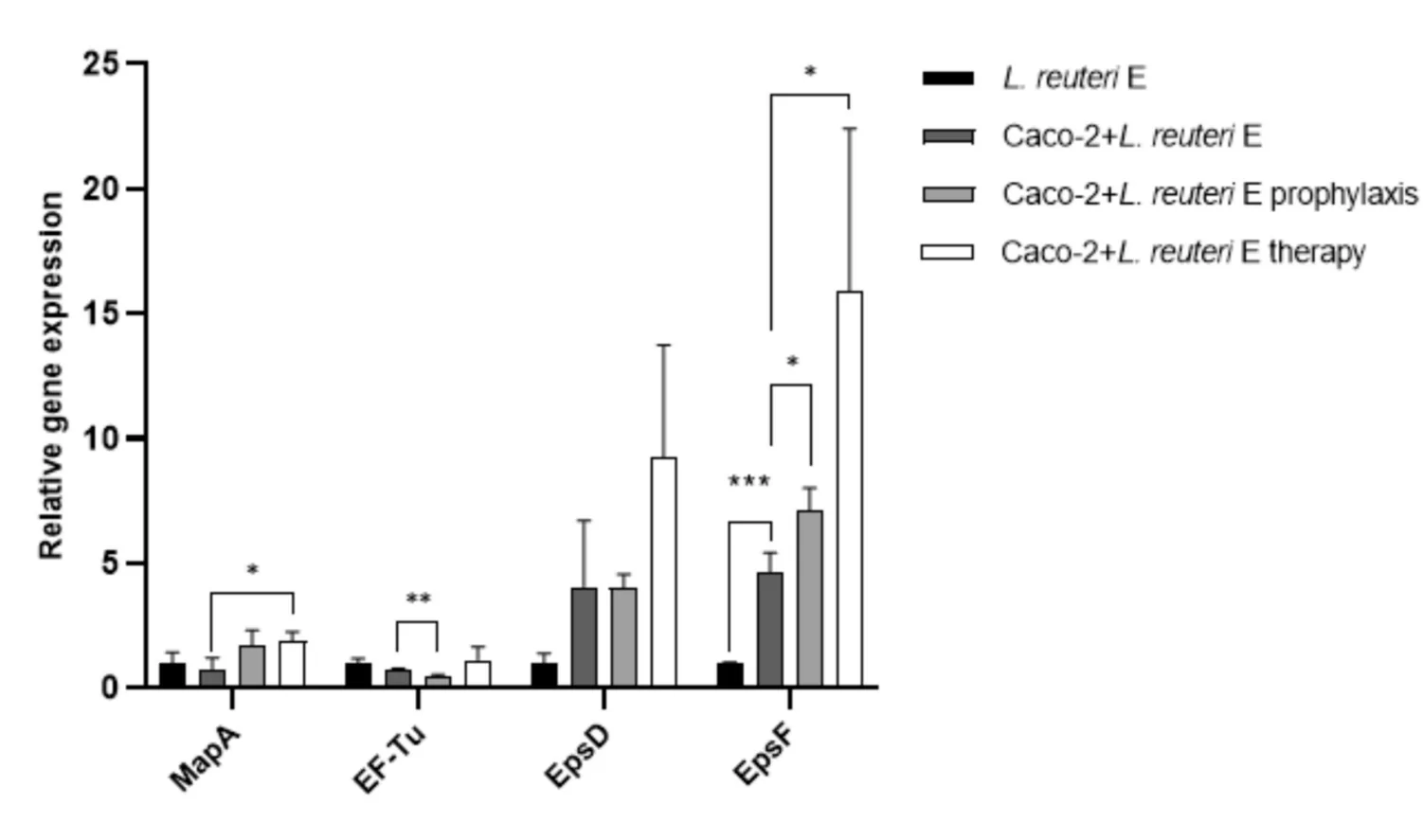
Expression of *L. reuteri* E genes encoding proteins involved in exopolysaccharide synthesis (EpsD, EpsF) and adhesion surface proteins (MapA, EF-Tu) in prophylaxis and therapy of 5-FU-treated Caco-2 cells. Data represent mean ± SD (*n* = 3). EF-Tu, elongation factor Tu; EpsD, tyrosine-protein kinase; EpsF, exopolysaccharide biosynthesis glycosyltransferase; MapA, mucus adhesion-promoting protein A; * *p* < 0.05, ** *p* < 0.01, *** *p* < 0.001

Adhesion of probiotics to the intestinal epithelium is necessary to achieve their specific therapeutic effects and bacterial colonization (Dudík et al. 2020). MapA, a part of ABC transporter in *L. reuteri* E, and elongation factor EF-Tu are so-called moonlighting proteins, bacterial multifunctional proteins. For example, EF-Tu and glycolytic pathway enzyme glycerol-3-aldehyde dehydrogenase were proven also to be involved in bacterial adhesion in *Bifidobacterium longum* and *Bacteroides thetaiotaomicron* (Nishiyama et al. 2023). MapA as lactobacillus adhesion factor was shown to bind receptor-like molecule paralemmin (PRLM) expressed on Caco-2 cells surface (Matsuo et al. 2012). According to MSA (multisequence alignment) of annotated genes and their corresponding PCR product sequences (data unpublished), we localized MapA as ABC transporter, substrate-binding protein (cluster 3, basic aa/glutamine/opines) in *L. reuteri* E WGS (Supplemental Table S1). MapA expression was significantly up-regulated in therapy mode, while EF-Tu was down-regulated in prophylaxis (Fig. 2). As EF-Tu is involved in several cell processes, it is possible that these changes are not related to the adhesion process.

EPS are produced by bacterial cells as a protective and adhesion factor and as a factor involved in inter- and intra-microbial interactions (Di Cagno et al. 2006; Mayer et al. 2020). EpsF, an exopolysaccharide glycosyltransferase, is generally responsible for carbohydrate chain elongation by catalyzing the formation of glycosidic linkages between sugar residues. In lactic acid bacteria, EpsD (tyrosine-protein kinase) forms a protein complex with EpsC (tyrosine-protein kinase transmembrane modulator) responsible for determining chain length and secreting complete mature EPS (Bentley et al. 2006; Leeber et al. 2009). In our experiments, we did not observe a significant change in EpsD expression. However, an increasing trend towards up-regulation was observed in the therapy model (Fig. 2). In the case of EpsF, we observed a significantly increased expression after co-cultivation with untreated Caco-2 cells (Fig. 2; *p* < 0.001). Furthermore, significant upregulation of the EpsF gene was observed in both the prophylaxis and therapy models. This may indicate that the immunomodulatory potential of *L. reuteri* E acts through the production of EPS, mainly EpsF. EpsF may play a role in intercellular communication between intestinal Caco-2 cells and lactobacilli.

**Fig. 3:**
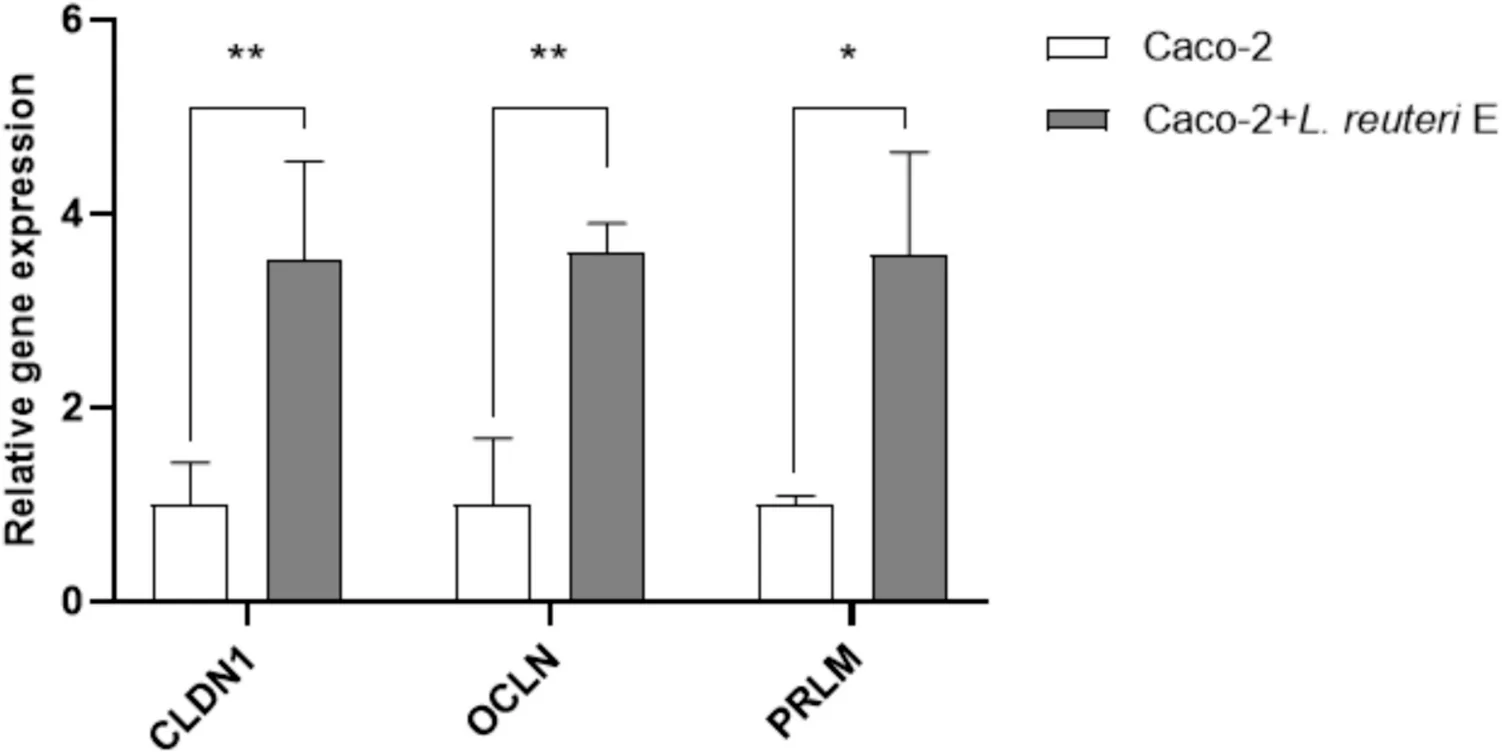
Effect of *L. reuteri* E on the gene expression of TJ proteins (CLDN1, OCLN) and receptor-like molecule paralemmin in Caco-2 cells. Data represent mean ± SD (*n* = 3). CLDN1, claudin 1; OCLN, occludin; PRLM, paralemmin; * *p* < 0.05, ** *p* < 0.01

**Fig. 4:**
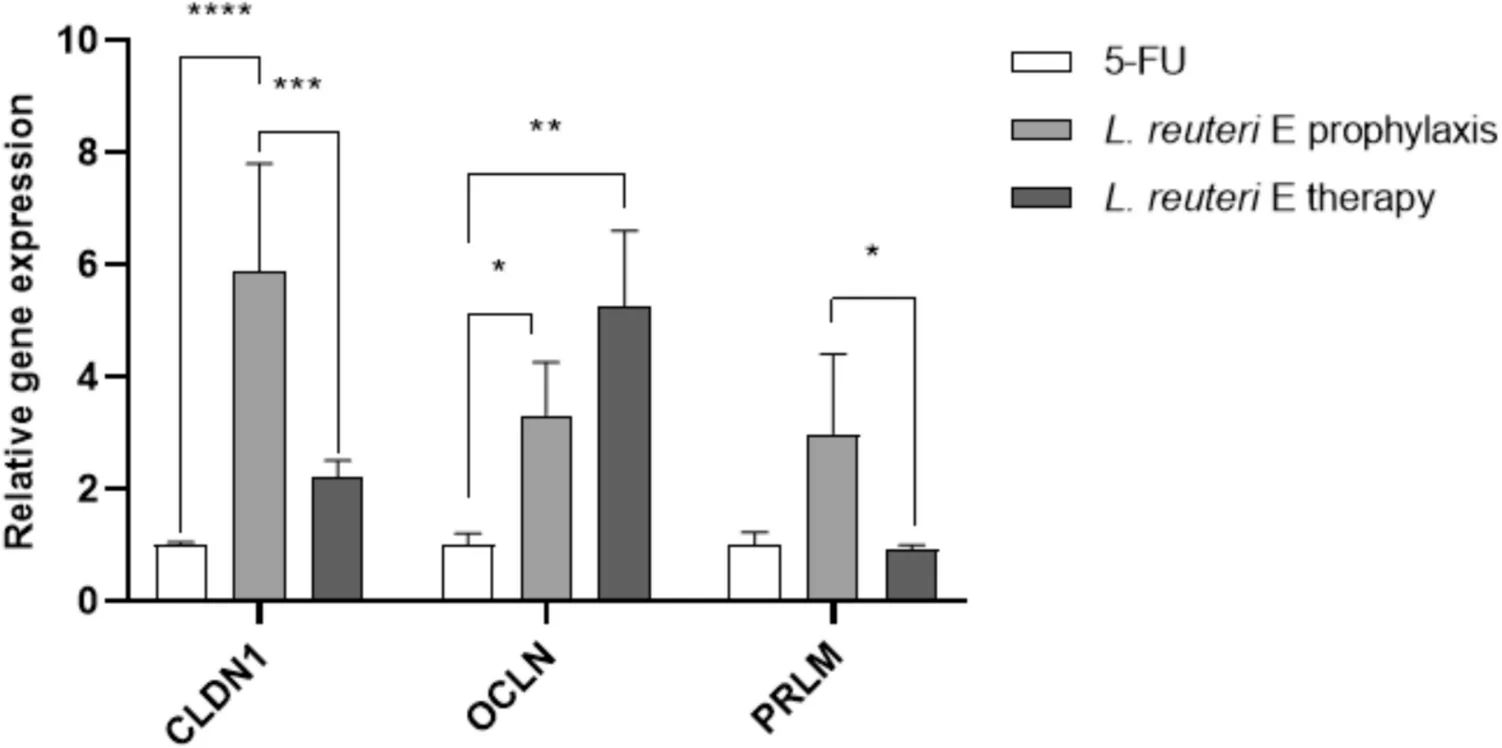
Prophylactic and therapeutic effect of *L. reuteri* E on the gene expression of TJ proteins (CLDN1, OCLN) and receptor-like molecule paralemmin in 5-FU-treated Caco-2 cells. Data represent mean ± SD (*n* = 3). CLDN1, claudin 1; OCLN, occludin; PRLM, paralemmin; * *p* < 0.05, ** *p* < 0.01, *** *p* < 0.001, **** *p* < 0.0001

Singh et al. (2021) described enhanced expression of MapA in six *L. reuteri* strains and its eukaryotic receptor-like molecules annexin A13 and PRLM after co-cultivation with Caco-2 cells. Matsuo et al. (2013) showed that the middle region of protein MapA bears the adhesion role to PRLM in contrast to annexin A13 and supposed that both receptor-like molecules work through different mechanisms. In our experiment, co-cultivation of *L. reuteri* E with untreated Caco-2 increased expression of PRLM (Fig. 3), while its lactobacilli ligand MapA was significantly up-regulated only in bacteria administered in therapy of 5-FU-treated Caco-2 cells (Fig. 2). On the contrary, in 5-FU-treated Caco-2 cells, *L. reuteri* E changed the expression of PRLM neither in prevention nor therapy. However, PRLM was up-regulated in prophylactic *L. reuteri* E administration when compared to therapy (Fig. 4).

The appropriate function of the intestinal lining is crucial for dysbiosis prevention and maintaining gut health (Mu et al. 2017). It was shown that intestinal bacteria regulate intestinal barrier function by changing the expression of TJ proteins (Buckley and Turner 2018). *L. reuteri* E increased gene expression of TJ proteins claudin 1 and occludin in healthy Caco-2 cells after co-cultivation (Fig. 3). According to Wang et al. (2022), *L. reuteri* I5007 increased the expression of *zonula occludens* 1, CLDN1, and OCLN in jejunal and ileal mucosa of suckling piglets.

In Caco-2 cells exposed to 5-FU, the effect of *L. reuteri* E treatment on TJ gene expression differed depending on whether bacteria were applied as prevention or therapy (Fig. 4). CLDN1 was significantly up-regulated only in prevention. On the other side, OCLN was up-regulated in both prevention and therapy with lactobacilli compared to 5-FU-treated Caco-2 control. Isolated bacterial EPS have been shown to up-regulate the expression of TJs *zonula occludens* 1, CLDN1, and OCLN in epithelial cells (Zhou et al. 2018; Chen et al. 2019). We observed increased expression of the two TJs studied, OCLN and CLDN1, when Caco-2 were co-cultivated with lactobacilli (Fig. 3) and in prophylaxis of 5-FU treatment (Fig. 4). At the same time, EpsF was up-regulated in *L. reuteri* E (Fig. 2). Our results confirm the direct correlation between gene expression of EpsF and TJs.

**Fig. 5:**
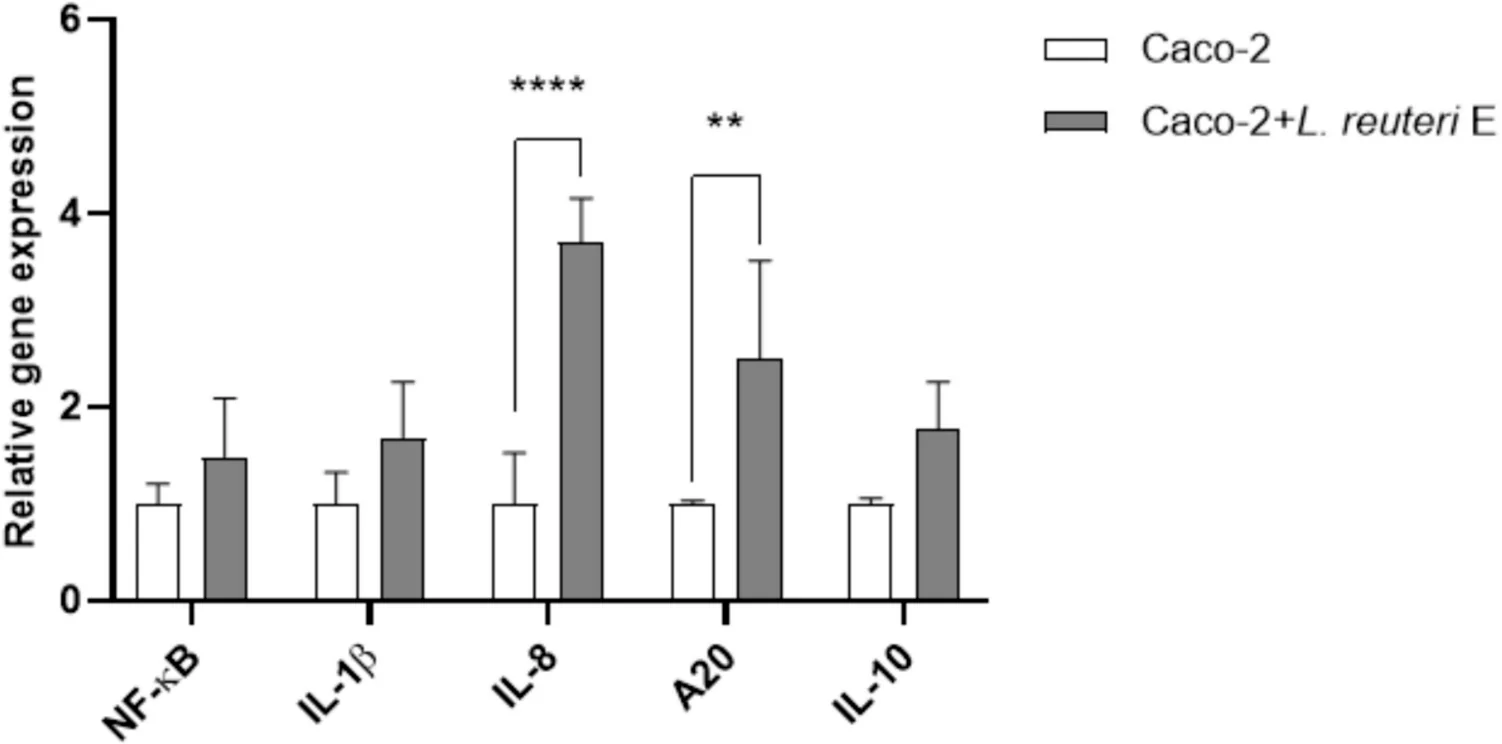
Effect of *L. reuteri* E on selected immune response genes expression in Caco-2 cells. Data represent mean ± SD (*n* = 3). A20, endogenous negative regulator of NF-κB; IL-1β, interleukin 1β; IL-8, interleukin 8; IL-10, interleukin 10; NF-κB, nuclear factor κB; ** *p* < 0.01, **** *p* < 0.0001

The other point we focused on was the ability of the investigated bacterial strain to impact pro- and anti-inflammatory markers in Caco-2 cells when used in prophylaxis or therapy of 5-FU treatment. The pro-inflammatory NF-κB pathway is activated by binding of a specific ligand to pattern recognition receptors (PRRs), which represent an important part of innate anti-infective immunity. An appropriate microbial ligand may activate different pro- or/and anti-inflammatory processes. Bacterial EPS have various effects on inflammatory pathways in intestinal cells. For example, in porcine intestinal cells, different bifidobacterial strains showed ability to inhibit the NF-κB pathway and its cytokines through up-regulation of A20 expression (Tomosada et al. 2013). The MAMP (microbe-associated molecular patterns)-activated NF-κB inflammatory pathway is tightly controlled by the zinc finger regulatory enzyme A20 (also called tumour necrosis factor-α-induced protein 3; TNFAIP3), which is involved in ubiquitination/deubiquitination, an important type of post-translational protein modification (Wertz et al. 2004; Das et al. 2018). Our results showed that *L. reuteri* E significantly increased gene expression of pro-inflammatory NF-κB pathway cytokine IL-8 and simultaneously increased A20 mRNA levels in Caco-2 cells (Fig. 5). This may indicate a negative regulation of this pathway since, at the same time, NF-κB expression is unchanged. Li et al. (2014) proved that *Lactobacillus acidophilus* NCFM EPSs significantly up-regulated NF-κB and p38 MAPK (mitogen-activated protein kinase) pathways in Caco-2 cells and suggested its potential immunoregulatory mechanism. Also, other bacterial structures, e.g., lipid chains of lipoteichoic acids, may induce expression of IL-8 in epithelial cells, such as intestinal Caco-2 or pulmonary A549 lines (Claes et al. 2012; Noh et al. 2015; Woo 2020). This points to different functions of bacterial surface structures in the regulation of immune response mechanisms.

**Fig. 6:**
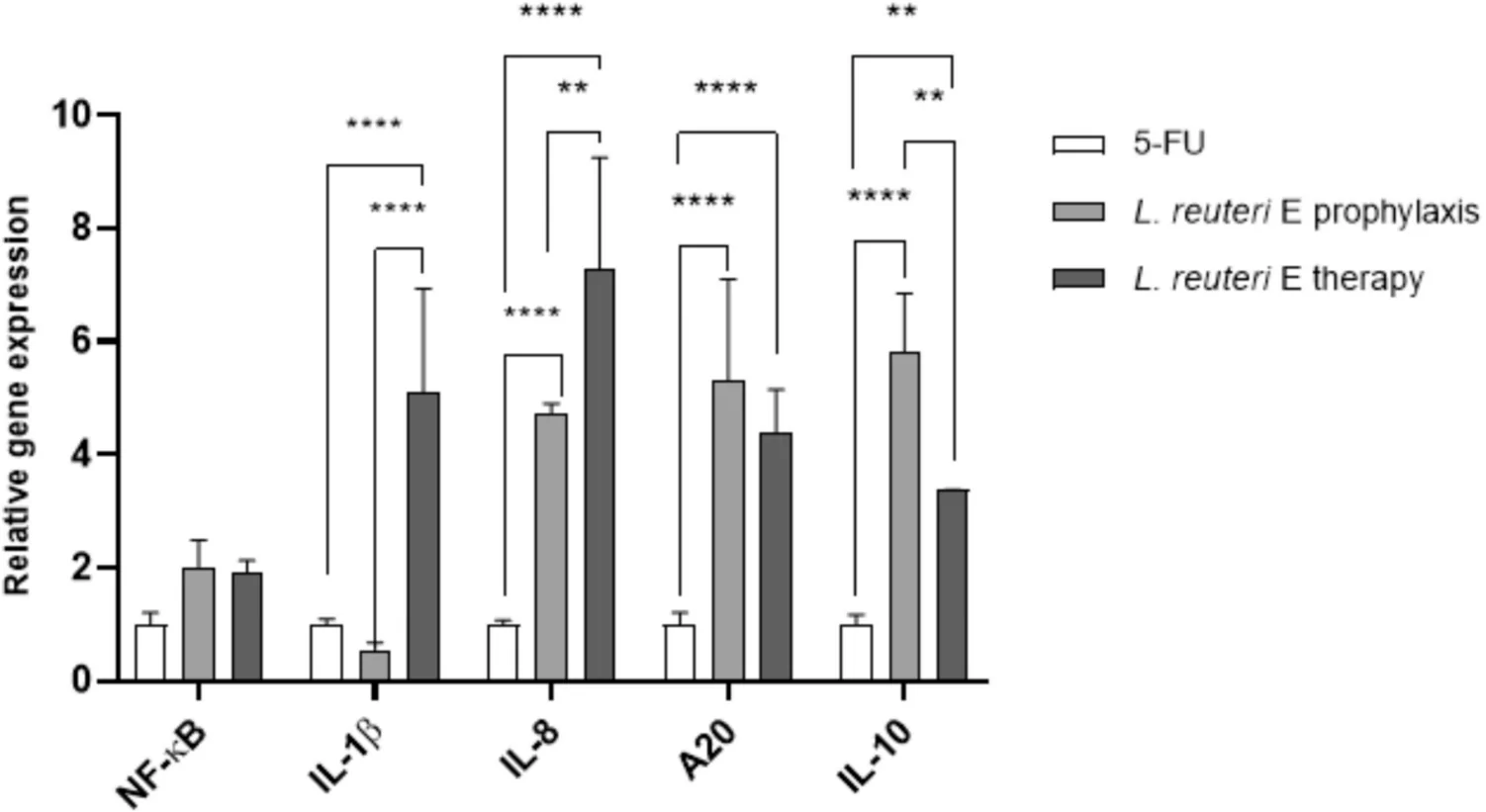
Prophylactic and therapeutic effect of *L. reuteri* E on 5-FU-treated Caco-2 cells. Data represent mean ± SD (*n* = 3). A20, endogenous negative regulator of NF-κB; IL-1β, interleukin 1β; IL-8, interleukin 8; IL-10, interleukin 10; NF-κB, nuclear factor κB; ** *p* < 0.01, **** *p* < 0.0001

Finally, we investigated the effect of *L. reuteri* E as a potential protective and/or therapeutic probiotic in 5-FU-induced intestinal mucositis in vitro. 5-FU has been shown to induce mucositis through activation of the NF-κB pathway (Chang et al. 2012). Although *L. reuteri* E did not significantly alter the gene expression of NF-κB in any experimental setting, IL-1β and IL-8, the cytokine and chemokine of this inflammatory pathway, were up-regulated after administration of lactobacilli as treatment of 5-FU damaged Caco-2 (Fig. 6). This suggests that lactobacilli potentiate the effect of 5-FU on Caco-2 cells under the set conditions. The prophylactic use of *L. reuteri* E may serve as a protective factor in the induction of inflammation through NF-κB and consequently IL-1β, both of which were unchanged. However, we observed an increased expression of IL-8, which is up-regulated via TLRs in response to bacterial MAMPs. The IL-10 and A20 can inhibit both pro-inflammatory cytokines, IL-1β and IL-8, through inhibition of NF-κB (Shembade et al. 2010; Sun et al. 2019; Carlini et al. 2023). Both of these anti-inflammatory factors, A20 and IL-10, were up-regulated in lactobacilli-treated Caco-2 cells (Fig. 6). This may represent the feedback response to increased mRNA expression of IL-1β (therapy) and IL-8 (both prophylaxis and therapy).

On the other side, the stimulation of pro-inflammatory cytokines IL-1β and IL-8 may be the immune response to increased gene expression of bacterial EpsF (Fig. 2) and eventual production of immunomodulatory exopolysaccharides.

Interestingly, when comparing the gene expression of pro- and anti-inflammatory cytokines, a trend of higher up-regulation of pro-inflammatory genes (IL-1β and IL-8) than anti-inflammatory ones (A20 and IL-10) was observed in *L. reuteri* E therapy of 5-FU-treated Caco-2 (Fig. 6). The opposite trend was observed in the prophylaxis mode, where anti-inflammatory genes were up-regulated more than pro-inflammatory ones. These differences between the prophylactic and therapeutic models may explain the results of the viability assay. The reduction in Caco-2 cell viability observed in the therapeutic setting (Fig. 1) may be partially due to an exacerbated inflammatory response triggered by *L. reuteri* E, as shown by the increased expression of IL-8 and IL-1β (Fig. 6). The increased levels of the anti-inflammatory regulators A20 and IL-10 may not have been sufficient to counterbalance the pro-inflammatory signaling in the already stressed epithelial cells. This suggests that, while the strain’s immunomodulatory activity may be beneficial in healthy or pre-treated cells, it may become detrimental in a damaged inflammatory microenvironment.

However, one of the limitations of this study is that inflammatory responses were assessed solely at the level of gene expression. While the up-regulation of IL-8 and IL-1β mRNA suggests activation of pro-inflammatory pathways—particularly in the therapeutic setting after 5-FU treatment—mRNA levels do not necessarily reflect actual cytokine production or secretion. Post-transcriptional regulation, translational control, and protein stability may significantly influence the final inflammatory output. Therefore, future studies should include protein-level analyses (e.g., ELISA or multiplex cytokine assays) to confirm whether transcriptional changes result in biologically relevant cytokine release and to better understand the impact of *L. reuteri* E on epithelial inflammation and viability.

## Conclusion

In conclusion, we can summarize that the potentially probiotic strain *L. reuteri* E shows the ability to modulate inflammation in Caco-2 cells induced by the chemotherapeutic agent 5-FU, possibly by the production of exopolysaccharides mediated by glycosyltransferase EpsF. Overall, according to the viability and gene expression of inflammatory markers A20, IL-10, IL-1β, and IL-8 in 5-FU-treated Caco-2 cells, it seems that *L. reuteri* E administration is more effective in prophylaxis than in therapy. Furthermore, the prophylactic use of lactobacilli could strengthen the intestinal barrier by increasing the expression of the TJ proteins claudin 1 and occludin. This study focused on the in vitro properties of *L. reuteri* E; however, its prophylactic use in IM requires further studies at the protein level as well as in vivo experimentation. Based on our results, probiotic application appears to be more effective prior to the onset of IM. However, further research is needed to shed more light on this issue.

Future research on *L. reuteri* E in well-designed clinical trials and other disease models, such as inflammatory bowel disease and immunocompromised conditions, may provide new insights into the effects of this strain. Such studies would validate its prophylactic and/or therapeutic potential and define appropriate dosing strategies for clinical use.

## Supplementary Information

Below is the link to the electronic supplementary material.

Supplementary file1 (DOCX 22.4 KB)

## Data Availability

The data supporting the findings are available within the article.
