# Supplementary material for: Prophylactic and therapeutic effects of Limosilactobacillus reuteri E on 5-fluorouracil-induced intestinal mucositis in Caco-2 cells
Source: Folia Microbiol (Praha). 2025 Aug 28;71(5):1193–204. doi: 10.1007/s12223-025-01310-3 (PMC13736486; doi:10.1007/s12223-025-01310-3)
Supplement: Supplementary file 1 [file 12223_2025_1310_MOESM1_ESM.docx]

***Limosilactobacillus reuteri* E is prone to improve viability of 5-fluorouracil damaged Caco-2 cells through up-regulation of immunomodulatory molecules**

**Folia Microbiologica**

Barbora Hlubinová, Hana Kiňová Sepová^*^, Ľudmila Pašková, Peter Novák, František Bilka, Hana Drahovská, Tomáš Szemes, Andrea Bilková

^*^Department of Cell and Molecular Biology of Drugs, Faculty of Pharmacy, Comenius University Bratislava, 831 41 Bratislava, Slovak Republic; [kinovasepova@fpharm.uniba.sk](mailto:kinovasepova@fpharm.uniba.sk)

**Supplemental** **Table S1** Characterization of selected *L. reuteri* E genes. Gene expression of coloured genes was researched in present study

|  | **Product** | **Gene Symbol** | **Contig** | **Accession** | **Start** | **End** | **Strand** | **Sequence length (nucleotides)** | **Sequence length (amino acids)** |
| --- | --- | --- | --- | --- | --- | --- | --- | --- | --- |
| Adhesion | Translation elongation factor Tu | tuf | 4 | JAHQZV010000004 | 15229 | 16419 | - | 1191 | 396 |
|  | ABC transporter, substrate-binding protein (cluster 3, basic aa/glutamine/opines) |  | 9 | JAHQZV010000009 | 40607 | 41398 | - | 792 | 263 |
| Virulence | Glucose-1-phosphate thymidylyltransferase (EC 2.7.7.24) | rfbA | 39 | JAHQZV010000039 | 1 | 828 | + | 828 | 275 |
|  | dTDP-glucose 4,6-dehydratase (EC 4.2.1.46) | rfbB | 39 | JAHQZV010000039 | 1435 | 2475 | + | 1041 | 346 |
|  | dTDP-4-dehydrorhamnose reductase (EC 1.1.1.133) | rfbD | 39 | JAHQZV010000039 | 2622 | 3461 | + | 840 | 279 |
|  | dTDP-4-dehydrorhamnose 3,5-epimerase (EC 5.1.3.13) | rfbC | 39 | JAHQZV010000039 | 842 | 1423 | + | 582 | 193 |
|  | dTDP-glucose 4,6-dehydratase (EC 4.2.1.46) | rfbB | 58 | JAHQZV010000058 | 1102 | 2142 | - | 1041 | 346 |
|  | dTDP-4-dehydrorhamnose reductase (EC 1.1.1.133) | rfbD | 58 | JAHQZV010000058 | 116 | 955 | - | 840 | 279 |
|  | dTDP-4-dehydrorhamnose 3,5-epimerase (EC 5.1.3.13) | rfbC | 58 | JAHQZV010000058 | 2154 | 2735 | - | 582 | 193 |
|  | Glucose-1-phosphate thymidylyltransferase (EC 2.7.7.24) | rfbA | 58 | JAHQZV010000058 | 2749 | 3618 | - | 870 | 289 |
| Exopolysaccharide production | Acyl-phosphate:glycerol-3-phosphate O-acyltransferase PlsY (EC 2.3.1.n3) | plsY | 2 | JAHQZV010000002 | 39165 | 39791 | + | 627 | 208 |
|  | Sugar kinase and transcription regulator (EC 2.7.1.-) |  | 5 | JAHQZV010000005 | 64306 | 65208 | + | 903 | 300 |
|  | Glycosyltransferase |  | 8 | JAHQZV010000008 | 825 | 2156 | - | 1332 | 443 |
|  | Phosphoenolpyruvate-protein phosphotransferase of PTS system (EC 2.7.3.9) | ptsP | 12 | JAHQZV010000012 | 8462 | 10189 | + | 1728 | 575 |
|  | L-O-lysylphosphatidylglycerol synthase (EC 2.3.2.3) | mprF | 16 | JAHQZV010000016 | 36775 | 39360 | - | 2586 | 861 |
|  | Glycosyltransferase |  | 20 | JAHQZV010000020 | 29628 | 30395 | + | 768 | 255 |
|  | Bactoprenol glucosyl transferase (EC 2.4.1.-); Glycosyltransferase, group 2 family protein |  | 20 | JAHQZV010000020 | 30413 | 31342 | + | 930 | 309 |
|  | Undecaprenyl-phosphate galactosephosphotransferase (EC 2.7.8.6) |  | 20 | JAHQZV010000020 | 31354 | 32001 | + | 648 | 215 |
|  | UDP-galactose:(galactosyl) LPS alpha1,2-galactosyltransferase WaaW (EC 2.4.1.-) |  | 22 | JAHQZV010000022 | 4289 | 5137 | + | 849 | 282 |
|  | Alpha-L-Rha alpha-1,3-L-rhamnosyltransferase (EC 2.4.1.-) |  | 27 | JAHQZV010000027 | 12056 | 12982 | - | 927 | 308 |
|  | Alpha-L-Rha alpha-1,3-L-rhamnosyltransferase (EC 2.4.1.-) |  | 27 | JAHQZV010000027 | 13989 | 14948 | - | 960 | 319 |
|  | UDP-galactopyranose mutase (EC 5.4.99.9) | glf | 27 | JAHQZV010000027 | 16405 | 17523 | - | 1119 | 372 |
|  | Glycosyltransferase |  | 27 | JAHQZV010000027 | 3866 | 4816 | - | 951 | 316 |
|  | Low molecular weight protein tyrosine phosphatase (EC 3.1.3.48) |  | 36 | JAHQZV010000036 | 7077 | 7541 | + | 465 | 154 |
|  | Manganese-dependent protein-tyrosine phosphatase (EC 3.1.3.48) |  | 37 | JAHQZV010000037 | 9313 | 10083 | + | 771 | 256 |
|  | Fructosamine-3-kinase (EC 2.7.1.-) |  | 41 | JAHQZV010000041 | 2321 | 3169 | + | 849 | 282 |
|  | Phosphotransferase system, phosphocarrier protein HPr |  | 43 | JAHQZV010000043 | 6545 | 6811 | + | 267 | 88 |
|  | Phosphoenolpyruvate-protein phosphotransferase of PTS system (EC 2.7.3.9) | ptsP | 43 | JAHQZV010000043 | 6811 | 8541 | + | 1731 | 576 |
|  | Glycosyltransferase LafA, responsible for the formation of Glc-DAG |  | 43 | JAHQZV010000043 | 8775 | 9992 | + | 1218 | 405 |
|  | Glycosyltransferase LafB, responsible for the formation of Gal-Glc-DAG |  | 43 | JAHQZV010000043 | 9995 | 11023 | + | 1029 | 342 |
|  | Exopolysaccharide biosynthesis glycosyltransferase EpsF (EC 2.4.1.-) |  | 47 | JAHQZV010000047 | 753 | 1856 | + | 1104 | 367 |
|  | Glucose-1-phosphate thymidylyltransferase (EC 2.7.7.24) |  | 47 | JAHQZV010000047 | 9482 | 9577 | + | 96 | 32 |
|  | Cell envelope-associated transcriptional attenuator LytR-CpsA-Psr, subfamily F2 |  | 54 | JAHQZV010000054 | 2092 | 3561 | - | 1470 | 489 |
|  | Cell envelope-associated transcriptional attenuator LytR-CpsA-Psr, subfamily F2 |  | 57 | JAHQZV010000057 | 1723 | 2724 | - | 1002 | 333 |
|  | Tyrosine-protein kinase EpsD (EC 2.7.10.2) |  | 57 | JAHQZV010000057 | 63 | 815 | - | 753 | 250 |
|  | Tyrosine-protein kinase transmembrane modulator EpsC |  | 57 | JAHQZV010000057 | 828 | 1703 | - | 876 | 291 |
